# Specific Frequency Electroacupuncture Stimulation Transiently Enhances the Permeability of the Blood-Brain Barrier and Induces Tight Junction Changes

**DOI:** 10.3389/fnins.2020.582324

**Published:** 2020-10-06

**Authors:** Shanshan Zhang, Peng Gong, Jiangsong Zhang, Xuqing Mao, Yibin Zhao, Hao Wang, Lin Gan, Xianming Lin

**Affiliations:** Department of Neurobiology and Acupuncture Research, The Third Clinical Medical College, Zhejiang Chinese Medical University, Key Laboratory of Acupuncture and Neurology of Zhejiang Province, Hangzhou, China

**Keywords:** electroacupuncture, blood-brain barrier, neurovascular unit, tight junction, frequency

## Abstract

The blood-brain barrier (BBB) plays an important role in both the physiological state and pharmacological state of the brain. Transiently enhancing the permeability of the BBB may allow the use of more types of medications for neuropsychiatric diseases. Our previous research revealed that electroacupuncture (EA) stimulation at certain parameters can enhance the permeability of the BBB in Sprague-Dawley rats, but this phenomenon is not well characterized. We propose that specific frequency EA can transiently open the BBB and may be related to the change of tight junctions (TJ). To find the best EA frequency among commonly used frequencies, preliminarily explore the mechanism, we detected BBB permeability by measuring the intensity of Evans Blue and 20 kDa FITC-dextran fluorescence in the cerebral cortex. Then, we used a laser spectrometer, immunofluorescence, western blotting, and transmission electron microscopy to detect the mechanism of BBB opening. Finally, measured brain water content, AQP4, GFAP, Iba1, and used the DeadEnd^TM^ Fluorometric TUNEL System to clear whether the stimulation caused obvious negative effects. The results show that EA stimulation at 2/100 Hz maximally increased BBB permeability, and the BBB closed within 12 h after EA stimulation was removed. EA stimulation increased blood perfusion, c-fos levels, and Substance P expression in the cerebral cortex, decreased ZO-1 and occludin levels and induced ultrastructural changes in TJ morphology. EA stimulation at specific parameters did not cause brain edema, activation of glial cells, or cell apoptosis. This study shows that EA stimulation induces a reversible, frequency-dependent alteration of BBB permeability and proposes a hypothetical mechanism of BBB opening related to vasodilation and TJ disruption. Transiently enhancing the permeability of the BBB with EA at specific parameters may be a new strategies for delivering therapeutics to the central nervous system. Further study of this technology is needed.

## Introduction

The blood-brain barrier (BBB), mainly composed of brain capillary endothelial cells, tight junctions (TJ), pericytes, basement membrane, and astrocytes foot processes (Liebner et al., 2011), prevents undesirable substances from entering the brain while allowing entry of necessary compounds (Banks, 2009). It is not a passive barrier but can change based on the demands of the central nervous system (CNS). The structure responsible for this plasticity is known as the neurovascular unit (NVU) (Banks and Erickson, 2010). Because of its structure, the BBB impedes the delivery of many macromolecular drugs to the CNS, and experimental protocols for selectively modulating the NVU to allow the delivery of therapeutic drugs through the BBB to treat neurological diseases are being investigated (Neuwelt et al., 2008; Dong, 2018). For example, prodrugs that can passively diffuse into cells through the BBB, drug conjugates for receptor-mediated transport through the BBB, liposome-mediated brain delivery, brain delivery of nanoparticles, exosome-mediated brain delivery, delivery of viral vectors to the brain (Vykhodtseva et al., 2008; Sahni et al., 2011; Liu et al., 2012; Vieira and Gamarra, 2016) via paracellular pathways through increases in the opening or porosity of tight intercellular junctions, like osmotic delivery (Bellavance et al., 2008) and peptide derivatives that can modulate the TJ of the BBB for a short period (Ulapane et al., 2017) are being studied. However, some of these methods of opening the BBB are challenging, some are difficult to apply in the clinic, and some are still in the experimental stage. Therefore, exploring applicable methods to effectively, non-invasively, and reversibly enhance the permeability of the BBB to facilitate drug delivery to the brain is of great clinical interest and is an important pursuit.

Electroacupuncture (EA), which is a modern way of administering acupuncture, refers to the application of a pulsating electrical current to acupuncture needles for acupuncture point stimulation. Because the stimulatory parameters of EA can be easily and clearly controlled, it has been commonly used in clinical therapy and basic acupuncture research (Fang et al., 2017). EA therapy is a common method for treating CNS diseases in the clinic (Tsui and Leung, 2002). The GV20 (Baihui) and GV26 (Shuigou) acupoints are located on the head and face and have the functions of refreshing and calming the mind. Modern studies have shown that GV20 and GV26 have a regulatory effect on the CNS and can alleviate cerebral nerve disorders, improve cerebral vascular circulation and increase cerebral blood flow; thus, these acupoints can be widely used for brain-derived diseases (Lv and He, 2016; Ran et al., 2019). We believe that EA at GV20 and GV26 can be used to treat CNS diseases, possibly due to its ability to enhance the permeability of the BBB and promote the entry of beneficial substances into brain tissue. Earlier, we found that acupuncture point injection of camphol and EA can effectively enhance the permeability of the BBB to Evans blue (EB) (Lin, 2007).

Notably, EA can have complex effects on the CNS that are mediated by multiple factors, such as the acupuncture points stimulated, approach, and parameters. Our previous study showed that 100 Hz EA at GV20 and GV26 can increase the level of nerve growth factor (NGF) in the cerebral tissues of middle cerebral artery occlusion/reperfusion rats during the recovery period [3 weeks after the operation when the integrity of BBB was ensured (Abulrob et al., 2008)], favor the regeneration and repair of nerves, and did not cause cerebral edema or secondary brain injury (Zhang et al., 2018). But later we found that 100 Hz EA also did not have a very stable and best effect in enhancing the permeability of the BBB, so we systematically studied EA frequency to identify the best frequency for enhancing the permeability of the BBB. The reason that EA-induced BBB opening in rats did not cause obvious side effects remains to be elucidated. Thus, we evaluated the duration for which EA enhances the permeability of the BBB, determined whether this BBB opening is permanent or transient, when the BBB opens during EA stimulation, and when the BBB closes after the removal of EA stimulation. Subsequently, we conducted a preliminary study on the mechanism of EA opening of the BBB because clarifying how EA allows macromolecular substance in the blood vessels to enter the brain through the BBB can allow us to further optimize the parameters of EA to achieve precise control of the duration and location of BBB opening and thus provide a theoretical basis for the promotion of drug delivery to the CNS by EA. Our preliminary study prompted that the opening of the BBB by EA may be related to regulation of the NVU and a change in tight junction proteins expression.

Therefore, in this research, the effect of EA in enhancing BBB permeability and the underlying mechanism was studied. These experiments were conducted in two parts. First, we identified the optimal EA frequency for enhancing the permeability of the BBB to determine whether the effect of EA on the BBB is reversible. Second, based on the first part, the optimal parameters of EA were used to preliminarily explore the mechanism and clear whether it would cause obvious adverse reactions. The study would be beneficial to solve the difficult problem of delivering macromolecular drugs to brain tissue to treat neurological diseases.

## Materials and Methods

### Experimental Animals

All procedures in this study were performed in compliance with the National Institutes of Health Guide for Care and Use of Laboratory Animals. The experimental protocol was approved by the Institutional Animal Care and Use Committee of Zhejiang Chinese Medical University and conformed to internationally accepted ethical standards. All efforts were made to alleviate animal suffering, minimize the number of animals used, and utilize alternatives to *in vivo* techniques when possible. The experiments were performed on 3-month old male Sprague-Dawley rats. The animals were housed at 25 ± 2°C and 50 ± 10% humidity on a 12-h light/dark cycle. Food and water were accessible *ad libitum* and supplied by the Laboratory Animal Center of Zhejiang Chinese Medical University, Hangzhou, Zhejiang, China.

### Electroacupuncture Stimulation

The influence of different frequencies of EA on the degree of EB penetration in intact rats was investigated. Rats were randomly divided into one of the following 8 groups (*n* = 12 rats each) the control group and EA groups with different frequencies (2, 15, 30, 50, 100, 2/50, 2/100 Hz). The rats were injected with 2% EB (Sigma, E2129-10G) in saline via the caudal vein using an indwelling needle (2 ml/kg). Rats in the EA groups underwent insertion of acupuncture needles (Beijing Zhongyan Taihe Medical Instrument Co., Ltd., China); a 25-mm long, 0.13-mm diameter needle was inserted at GV20 (Baihui), and a 16-mm long, 0.07-mm diameter needle was inserted at GV26 (Shuigou). The needles were then stimulated using an acupuncture point nerve stimulator (HANS-200, Nanjing Jinsheng, Ltd., China) to an intensity of 3 mA for 40 min. The control group did not undergo EA treatment and only received an injection of 2% EB in saline via the caudal vein. All experimental operated between 6:30 am and 8:30 pm Beijing time. EB content of the brain tissues and Cerebrospinal fluid (CSF) was measured in 8 rats from each group, and 4 rats were used to observe EB expression by VIVS and fluorescence microscopy.

To measure how long the BBB permeability was significantly enhanced during EA stimulation procedure, rats were randomly divided into one of the following 7 groups (*n* = 12 rats each): the control, 5, 15, and 40 min, 1, 2, and 4 h groups. The control group was injected with EB, which was allowed to circulate throughout the brain for 40 min, and then sacrificed. The 5, 15, and 40 min, 1, 2, and 4 h groups were injected with EB, then underwent 3 mA, 2/100 Hz (the frequency of EA was the optimal frequency identified in the experiment) EA stimulation for 5, 15, and 40 min, 1, 2, and 4 h, respectively, and sacrificed after 40 min. EB content was measured in 8 rats from each group, and 4 rats were used to observe EB expression by fluorescence microscopy.

To test whether EA can reversibly enhance the permeability of the BBB, rats were randomly divided into one of the following 5 groups (*n* = 12 rats each): the control, EA, EA-0 h, EA-5 h, and EA-12 h groups. The control group was injected with EB, which was allowed to circulate throughout the brain for 40 min, and then sacrificed. The EA group was first injected with EB and then sacrificed after EA (3 mA, 2/100 Hz, 40 min). The animals in the EA-0 h, EA-5 h, and EA-12 h groups were injected with EB immediately after EA, 5 h after EA, and 12 h after EA, respectively, and sacrificed after 40 min. EB content was measured in 8 rats from each group, and 4 rats were used to observe EB expression by fluorescence microscopy.

We repeatedly verified whether EA can reversibility enhance BBB permeability with 1 mL 20 kDa FITC-dextran (Sigma, FD20, 20 mg/mL) being administered intravenously to rat. Rats were randomly divided into one of the following 3 groups (*n* = 4 rats each): the control, EA, and EA-12 h groups. The control group was injected with FITC-dextran, which was allowed to circulate throughout the brain for 40 min, and then sacrificed. The EA group was first injected with FITC-dextran and then sacrificed after EA (3 mA, 2/100 Hz, 40 min). The EA-12 h groups were injected with FITC-dextran 12 h after EA and sacrificed after circulated for 40 min. FITC-dextran expression was observed with a digital pathological section (fluorescence) scanning analyzer.

To detect the effect of EA on cerebral blood flow in rats, changes in cerebral blood perfusion before and after EA were observed in 4 rats with a laser speckle meter after a window was opened in the skull. The influence of EA on c-fos, SP, TJ protein expression in the brain tissue of the rats, BBB structure were detected. Rats were randomly divided into the control group, EA group, and EA-12 h groups (*n* = 14 rats each). The influence of EA on GFAP, Iba1, and AQP4 expression in the brain tissue of the rats, brain water content, and cell apoptosis were also detected. Rats were randomly divided into a control group and an EA group (*n* = 18 rats each). The control group did not receive EA, the EA group was treated with EA (3 mA, 2/100 Hz, 40 min) and sacrificed immediately, while the EA-12 h group was treated with EA and sacrificed after 12 h.

### Cerebrospinal Fluid Extraction

After EA, we use a modified method to extract CSF (Li and Ji, 2012), anesthesia was induced with 4% isoflurane (RWD, R510-22-4), 1–2% isoflurane mixed air to maintain anesthesia, the hair on the head and neck was shaved with an animal shaver, and the head of the rat was fixed. The head and body were placed at an angle of approximately 135°, and the skin was disinfected with iodophor. After the rat was fixed, the occipital crest was found and indicated with a marker, the gap in the muscle 3 mm below the occipital crest was selected as the needle insertion point, the triangular depression was identified, and the needle was held with a 1-mL syringe. The needle was inserted parallel to the body, and the needle tip was slowly advanced upward into the cisterna magna at a depth of approximately 0.5 cm. A total of 80–100 μL of cerebrospinal fluid was slowly drawn, the needle was quickly withdrawn, and the cerebrospinal fluid was transferred to a 200-μL EP tube, placed in liquid nitrogen to quickly freeze it, and stored at −80°C.

### Brain Perfusion

After EA, the rats were deeply anesthetized with sodium pentobarbital (50 mg/kg). The animals were then perfused with 0.9% saline through the left ventricle until colorless fluid was obtained from the right atrium. After decapitation, the brains were removed, placed in liquid nitrogen to quickly freeze them, moved to ***−***80°C, and stored. For immunohistochemistry, rats were subsequently perfused with 4% PFA. After the body and limbs of the rats were stiff, the animals were decapitated, and the brains were removed, placed in paraformaldehyde, and fixed for 24 h.

### Blood-Brain Barrier Permeability Assays

First, using a multifunctional microplate reader (SpectraMax M5, Molecular Devices Co., United States) to measure EB content, whole cerebral cortex samples were weighed and homogenized in 50% trichloroacetic acid to precipitate protein by sonication. The samples were then cooled for 30 min and centrifuged at 10,000 × *g* for 20 min at 4°C. The supernatants were obtained and diluted threefold with ethanol. The concentration of the tracer in the supernatant was measured at 620 nm and 680 nm for excitation and emission, respectively. The extravasated EB is expressed as μg/g of brain tissue based on a standard curve (80 μg/mL of EB dissolved in saline with eight serial dilutions). Then, using IVIS to observe EB expression in brain surface, the samples were placed in a small animal physiological signal telemetry device, IVIS was used to detect the intensity of EB fluorescence in the Cy5.5 channel. To observe EB by laser confocal microscopy, frozen 30-μm-thick brain slices with 3 mm between each slice were selected. Photographs were taken with a laser confocal microscope (LCM, Nikon Eclipse Ti) in the Cy5 channel. FITC-dextran expression of frozen 30-μm-thick brain slices was observed with a digital pathological section (fluorescence) scanning analyzer, the green fluorescence integrated optical density (FIOD) values of FITC-dextran in the cerebral cortex were measured using Image-Pro Plus software 6.0 and statistically analyzed (FIOD/area^∗^100). FIOD values were used as measures of the deposition of FITC-dextran in the brain tissue.

### Regional Cerebral Blood Flow Measurement

To detect changes in cerebral blood flow in brain tissues during EA, the rats were anesthetized with 4% isoflurane. Anesthesia was maintained with 1–2% isoflurane in air, and the head of the rat was fixed on a stereotaxic instrument. A thermometer was placed under the abdomen of the rat, and the rectal temperature was maintained at 37 ± 2°C. An animal shaver to use to shave the hair on the head, the skin was disinfected with iodine, the left scalp was cut, the skull was exposed, the surface of the skull was cleaned with 30% H_2_O_2_, and a skull drill was used to grind the skull until the blood vessels on the surface of the cerebral cortex were visible to the naked eye. Glycerin was applied to the skull to make the blood vessels clearer. After the rat awoke, a laser speckle meter (PeriCam PSI HR, PSIH-10005) was used to measure blood flow in the tissues of the left hemisphere, determine the region of interest, and detect the local cerebral blood perfusion before, during EA, and after EA.

### Immunofluorescence Staining

Brains were isolated and post-fixed in 4% PFA overnight at 4°C before preservation in 30% sucrose in PBS. Brains were sectioned coronally or sagittally at a thickness of 30 μm on a Leica CM1950 cryostat, and sections were stored at −20°C. Sections were blocked with 0.3% Triton X-100 and 5% goat serum in 0.01 M phosphate-buffered saline (PBS) in a 37°C water bath incubator for 1 h before incubation at 4°C overnight with primary antibodies at the following concentrations: rabbit anti-c-fos (1:200, Cell Signaling, 2250S), rabbit anti-SP (1:400, Abcam, ab67006), rabbit anti-ZO-1 (1:300, Invitrogen, 61–7300), rabbit anti-Occludin (1:300, Invitrogen, 71–1500), mouse anti-Claudin 5 (1:300, Invitrogen, 35–2500), mouse anti-GFAP-Cy3^TM^ (1:300, Sigma, C9205), rabbit anti-Iba1 (1:200, Huabio, ET1705-78) and rabbit anti-Aquaporin (1:200, Huabio, ER1903-87) diluted in PBS containing 5% goat serum and 0.3% Triton X-100. After being rinsed, the brain sections were incubated in an Alexa Fluor^TM^ Plus 647-conjugated goat anti-rabbit secondary antibody (1:400, Invitrogen, A32733) or Alexa Fluor^TM^ Plus 488-conjugated goat anti-mouse secondary antibody (1:400, Invitrogen, A32723) diluted in PBS containing 5% goat serum and 0.3% Triton X-100 at 37°C in a constant temperature water bath for 1 h. After secondary antibody incubation, the brain slices were washed, dried, and mounted with Fluoroshield Mounting Medium with DAPI. Light was avoided after addition of the secondary antibody. The expression of SP was observed with a digital pathology section (fluorescence) scanning analyzer. The red FIOD values of SP in the same area of the cerebral cortex were measured using Image-Pro Plus software 6.0 and statistically analyzed (FIOD/area^∗^100). The expression of others was observed with Zeiss Axio Imager M2.

### Western Blot Analysis

Briefly, 20 μg of cerebral cortex samples protein from each group was separated by SDS-PAGE and transferred to a polyvinylidene fluoride membrane, which was blocked with 5% non-fat milk. The membrane was incubated with the following primary antibodies in Tris–buffered saline Tween (TBST), pH 7.4, at 4°C overnight: HRP-conjugated rabbit beta-actin mAb (1:1000, Cell Signaling, 5125S), mouse anti-c-fos (1:2000, Invitrogen, 4700), rabbit anti-ZO-1 (1:1000, Invitrogen, 61–7300), rabbit anti-Occludin (1:1000, Invitrogen, 71–1500), mouse anti-Claudin 5 (1:1000, Invitrogen, 35–2500), mouse anti-GFAP (1:2000, Invitrogen, MA5-12023), rabbit anti-Iba1 (1:1000, Huabio, ET1705-78), and rabbit anti-Aquaporin (1:1000, Huabio, ER1903-87). The membrane was incubated with an HRP-conjugated goat anti-rabbit/mouse (H+L) secondary antibody (Bioker Biotechnology) diluted 1:5000 in TBST for 2 h at room temperature. The protein bands were viewed with the ImageQuant LAS 4000 system, and densitometry was quantified using ImageJ software.

### Transmission Electron Microscopy

The brains were removed and placed at 4°C on ice. Approximately 1-mm^3^ blocks of tissue from the prefrontal cortex were immediately placed in 2.5% glutaraldehyde solution, fixed at 4°C for 2∼4 h, and rinsed in 0.1 M phosphate buffer, pH 7.0, on a shaker 3 times for 15 min each. The tissues were placed in a 1% osmium acid solution for 1∼2 h and then rinsed with 0.1 M phosphate buffer, pH 7.0, on a shaker 3 times for 15 min each. The brain tissue blocks were dehydrated in gradient ethanol solutions (50, 70, 80, 90, and 95%; 15 min each), incubated with 100% ethanol for 20 min, and placed in pure acetone for 20 min. For gradient infiltration with an embedding agent, the tissues were incubated with acetone solution (V/V = 1/1) for 1 h and in acetone solution (V/V = 3/1) for 3 h and kept away from light at room temperature overnight. The tissue blocks were placed in the Leica EM UC 7 ultrathin microtome, ultrasectioned at a thickness of 70 nm. The tissues were stained with uranyl acetate 50% ethanol saturated solution for 15 min∼2 h and with lead citrate for 15 min. The structure of the BBB was observed by Transmission electron microscopy (TEM) (Hitachi H-7650).

### Brain Water Content Measurement

The brain water content was measured using a standard wet/dry weight method. Eight rats from the control group and eight rats from the EA group were used. Rats from the normal control group were left untreated and sacrificed, and the brain was removed after direct anesthesia, and rats in the EA group were sacrificed to brain collection after stimulation. Filter paper was used to absorb the water on the surface of the brain, the wet weight (WW) of the tissues was recorded, and then the tissues were placed in a constant temperature oven at 110°C. The brains were weighed every 4 h until the weight remained unchanged for three consecutive weights. This weight was recorded as the dry weight (DW) of the rat brain. Brain water content was calculated using the following equation: brain water content (%) = (wet weight-dry weight)/(wet weight) × 100.

### TUNEL Staining Detection of Cell Apoptosis in Rat Brain

Brain tissues were removed, fixed, and rinsed with 0.1 M PBS solution until the brains exhibited no irritating odor. The brains were sliced sagitally in a mold at a thickness of 4 μm, placed in a tissue embedding box, and soaked in 75% alcohol for 24 h. After dehydration in gradient ethanol solutions, the slices were placed at 55°C and dried. The slices were placed in xylene I for 5 min followed by xylene II for 5 min and then hydrated in gradient alcohol solutions. Proteinase K without DNase was added dropwise to the slices, and the slices were incubated in a 37°C incubator for 30 min and rinsed with PBS. TUNEL reaction solution (G3250, PROMEGA) was added dropwise to the slices, and the slices were incubated in a 37°C incubator in the dark for 1 h, rinsed with PBS, dried, and mounted. TUNEL staining was observed with a digital pathological section (fluorescence) scanning analyzer. The green FIOD values of TUNEL in the same area of the cerebral cortex were measured using Image-Pro Plus software 6.0 and statistically analyzed (FIOD/area^∗^100).

### Statistical Analysis

Data in graphs are expressed as means ± SEM. Statistical analyses were carried out using IBM SPSS 20.0. One-way ANOVA followed by Tukey’s *post hoc* test was used for comparison among groups ≥3. Two-tailed Student’s *t*-test was used for comparisons between two groups. Comparison is considered significantly different if the *p*-value < 0.05.

## Results

### Effect of Different Frequencies of EA in Enhancing BBB Permeability

**FIGURE 1:**
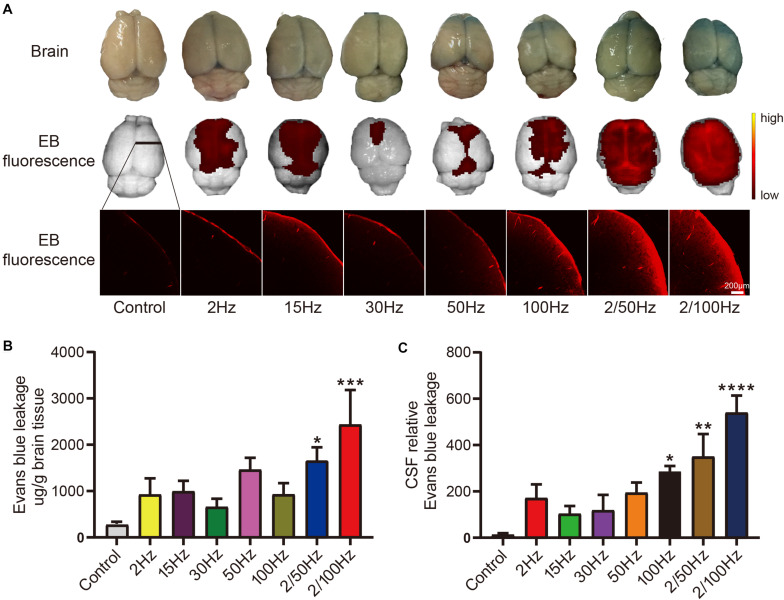
Effect of different frequencies of EA on the permeability of the BBB. **(A)** The first row shows the penetration of the BBB by EB in each group, which can be seen by the naked eye. The second row shows the fluorescence intensity and distribution of EB on the brain surface observed by IVIS in each group (Color Scale: Min = 2.25e^9^, Max = 2.96e^10^). The third row shows the fluorescence intensity and distribution of EB in coronal brain slices from each group. **(B)** EB content in the cerebral cortex in the groups subjected to EA at different frequencies and the control group; ***p < 0.001, 2/100 Hz vs control; *p < 0.05, 2/50 Hz vs control. **(C)** The relative content of EB in the cerebrospinal fluid in the groups subjected to EA at different frequencies and the control group; ****p < 0.0001, 2/100 Hz vs control; **p < 0.01, 2/50 Hz vs control; *p < 0.05, 100 Hz vs control; *n* = 8/group.

We detected the frequency of EA that induces the most robust enhancement of BBB permeability by detecting the level of EB penetration in rat cerebral cortex after EA at different frequencies. Naked eye observation (first row) of Figure 1A shows that the brain tissue was stained blue by EB to varying degrees. There was high EB content in the prefrontal lobe, while the EB content gradually decreased from the parietal lobe to the occipital lobe. IVIS (second row) shows the same trend of EB fluorescence as naked eye observation. Coronal brain slices (third row) show that EB was significantly increased in the cerebral cortex and that the fluorescence intensity was different between groups. Figure 1B shows EB penetration in rat cerebral cortex after EA at different frequencies. EB penetration in the 2/50 Hz group was higher than that in the control group (*p* < 0.05); EB penetration was increased in the 2/100 Hz group compared with the blank control group, and the difference was statistically significant (*p* < 0.001). Examination of EB content in the cerebrospinal fluid in Figure 1C shows that EB penetration in the 2/100 Hz group was significantly higher than that in the control group (*p* < 0.0001), EB penetration in the 100 Hz and 2/100 Hz groups was also increased compared to that in the control group (*p* < 0.05). The content of EB in the cerebral cortex and cerebrospinal fluid in the groups that received EA at other frequencies exhibited an increasing trend to different degrees compared with that in the control group, but there was no significant difference (*p* > 0.05). The differences in EB content in the cerebral cortex and cerebrospinal fluid suggested that different frequencies of EA have different effects on enhancing BBB permeability and that 2/100 Hz EA has a more robust effect on opening the BBB than EA at other frequencies.

### EA Can Stimulation-Dependently and Reversibly Open the BBB

**FIGURE 2:**
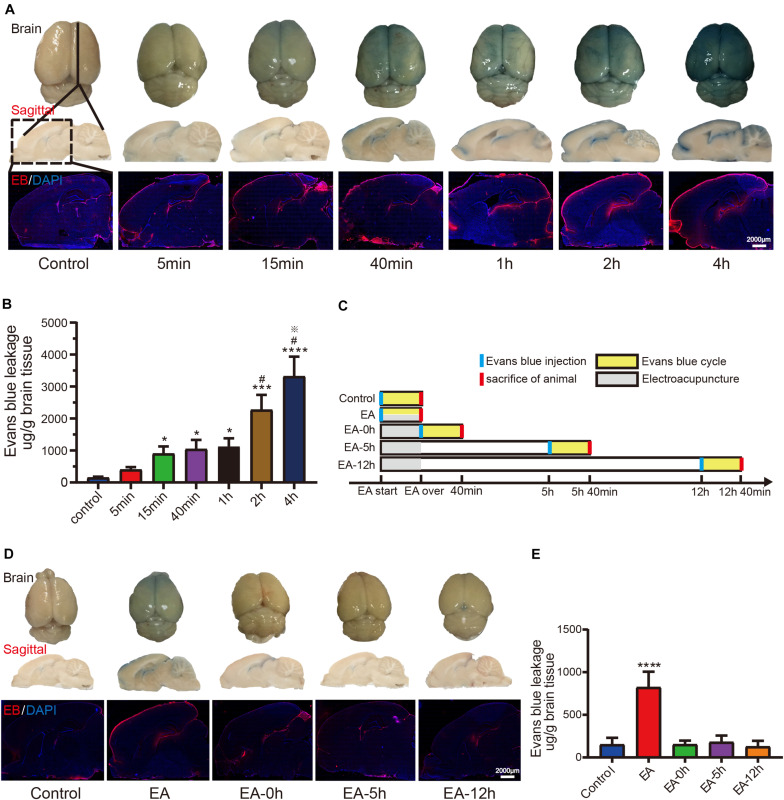
EA increases the permeability of the BBB, and this effect is stimulation-Dependently and reversibly**. (A)** The first row shows EB permeability in each group, which can be seen by the naked eye. The second row shows EB permeability in sagittal brain slices from each group, which can be seen by the naked eye. The third row shows the intensity and distribution of EB fluorescence in sagittal brain slices from each group. **(B)** EB content in the cerebral cortex in each group; *p < 0.05, 15 min/40 min/1 h vs control; ***p < 0.001, 2 h vs control; ****p < 0.0001, 4 h vs control; ^#^*p* < 0.05, 2 h/4 h vs 1 h; ^?^*p* < 0.05, 4 h vs 2 h; *n* = 8/group. **(C)** A flow chart showing the experimental protocol used to study the reversibility of EA-induced enhancement of BBB permeability. **(D)** The three rows show the intensity and distribution of EB in sagittal brain slices from each group. **(E)** EB content in the cerebral cortex in each group; ****p < 0.0001, EA vs control; *n* = 8/group.

After identifying the 2/100 Hz EA with the most robust effect on BBB opening, we used this frequency of EA to explore the duration of its effect in enhancing BBB permeability. First, we measured how long the BBB permeability was significantly enhanced during EA stimulation procedure. As Figure 2A shown, with the continuous stimulation of EA, EB penetration was more and more obvious in the brain surface (first row), Sagittal brain tissue sections (second row), and fluorescence (third row). By measuring the amount of EB penetration in the cerebral cortex of rats stimulated with different duration, as shown in Figure 2B, we found that the EB content in 15, 40 min, 1, 2, and 4 h groups were higher than the control group and the difference was statistically significant (*p* < 0.05), the EB content in 2 h group was higher than 1 h group and 4 h group was higher than 2 h group, the difference was statistically significant (*p* < 0.05). Then, we used 2/100 Hz EA for 40 min to detect whether the BBB remained permeable at three time points after EA stimulation. The specific protocol is shown in Figure 2C. In the EA-0 h, EA-5 h, and EA-12 h groups, EB was injected immediately, 5 and 12 h when EA over, respectively, and the animals were sacrificed after 40 min of circulation. Rats in the EA group were treated with EA after being injected with EB and sacrificed immediately after EA. The rats in the control group were sacrificed 40 min after EB injection. As seen in Figure 2D, EB penetration was obvious in the brain surface in the EA group (first row), and there were no obvious changes in the other groups. Sagittal brain tissue sections were observed (second row), and fluorescence image (third row) showed that there was more EB penetration in the cortex and around the lateral ventricle in the EA group than in the control group. Fluorescence image showed that there was little EB penetration in the EA-0 h group and EA-5 h group, but EB penetration was not observed in EA-12 h groups. By measuring the amount of EB penetration in the cerebral cortex of rats injected with EB at different time points before and after EA, as shown in Figure 2E, we found that the EB content in the EA group was significantly higher than the control group and that the difference was statistically significant (*p* < 0.01). The EA-0, EA-5, and EA-12 h groups exhibited no significant increase in EB content in brain tissue compared with that in the control group (*p* > 0.05). Figure 2 shows that 2/100 Hz EA for 40 min effectively enhanced the permeability of the BBB, it starts to have an obvious effect with 15 min stimulation and sustained EA stimulation leads to persistent BBB opening, but BBB closed after EA, and it completely closed within 12 h, thus the EA-induced enhancement of BBB permeability was stimulation-dependent and reversible.

### EA Activates Neurons to Alter Cerebral Blood Perfusion

**FIGURE 3:**
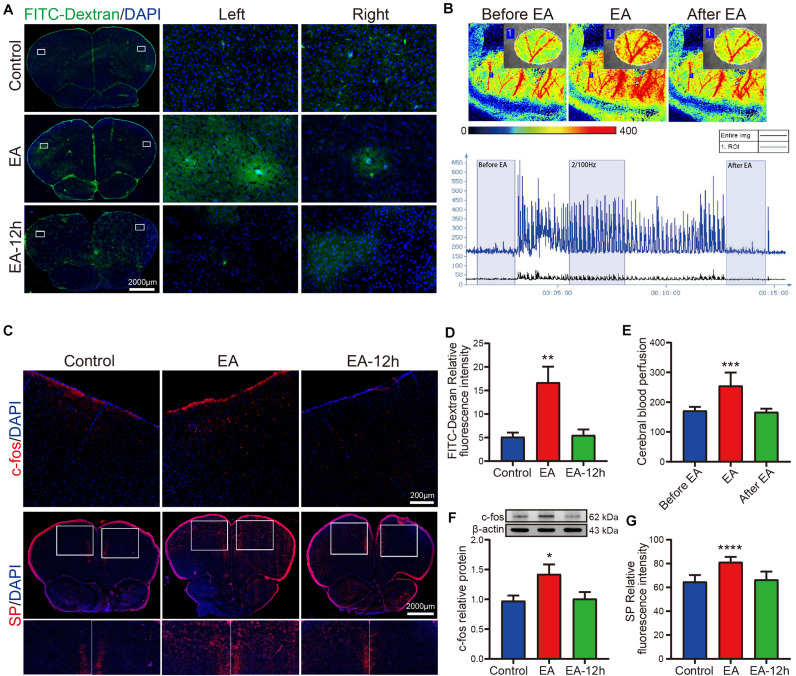
Effect of EA on cerebral blood perfusion, c-fos, and SP. **(A)** Fluorescence images showing that FITC-dextran diffused around the blood vessels in the EA group, control group, and EA-12 h group had no such phenomenon. **(B)** Heat spectrogram of cerebral blood flow changes in the ROI before and after EA, as measured by a laser speckle meter. Take a period of stationary period ROI-specific changes in cerebral blood flow perfusion for statistics. **(C)** Changes of c-fos and SP expression in the control, EA, and EA-12 h groups were observed by fluorescence microscope. **(D)** Statistical analysis of fluorescence intensity showing that FITC-dextran increased after EA; **p < 0.01, EA vs control; there was no significant difference between the control group and EA-12 h group; *n* = 4/group. **(E)** Cerebral blood flow perfusion was significantly increased during EA compared with before EA; ***p < 0.001, EA vs before EA; n = 4/group. **(F)** Western blot data show that c-fos expression increased in the EA group; *p < 0.05, EA vs control, *n* = 6/group. **(G)** Statistical analysis of fluorescence intensity showing that SP expression increased in EA group; ****p < 0.0001, EA vs control; *n* = 4/group.

We also verified EA can effectively and reversibly enhance the permeability of the BBB by 20 kDa FITC-dextran, and conducted a preliminary mechanistic study. As shown in Figure 3A, in the fluorescence images of brain tissue sections, we can see in the EA group many sheet-like FITC-dextrans are exuding from the blood vessels in the cortex, but the control and EA-12 h group can not see. The content of FITC-dextran in the EA group was significantly higher than the control group and the difference was statistically significant (*p* < 0.01), but the EA-12 h group had no significant increase compared to the control group (Figure 3D). To determine whether the permeability of the BBB induced by EA affects cerebral blood vessels, we used a laser speckle meter to observe cerebral blood flow during EA and selected a clear region of interest (ROI). The heat map showed that the perfusion of cortical blood vessels in the ROI was increased during EA and that the diameter of the blood vessels increased (Figure 3B). The amount of cerebral blood perfusion during EA was significantly increased compared to that before EA (*p* < 0.001), we also observed that after EA, the cerebral blood perfusion volume returned to the level observed before EA (Figure 3E). An increase in cerebral blood perfusion in the cortex is mainly related to the dilation of cerebral microvessels and arterioles, and we speculate that it may have been caused by changes to the structure of the NVU induced by EA. Therefore, we observed whether EA changed the activity of neurons in brain tissue and found that c-fos expression increased after EA. As shown in Figures 3C,F, the fluorescence image revealed that c-fos expression was increased in the cortex in the EA group and western blot analysis shown the increase was statistically significant (*p* < 0.05), but the EA-12 h group had no significant increase compared to control group. Previously, we learned that when neurons are activated, certain vasoactive substances can be released, resulting in increased blood perfusion when the blood vessels dilate. Finally, we found that the expression of the vasoactive substance SP was changed after EA and that the SP content in the EA group was significantly higher than that in the control group (*p* < 0.0001), EA-12 h group had no significant increase compared to the control group (Figures 3C,G). Therefore, EA stimulation can promote the activation of neurons in brain tissue, increasing the release of the vasoactive substance SP and vasodilation, the external manifestation is an increase in cerebral blood perfusion, and all changes disappear after EA over.

### EA Affects the Expression of Tight Junction Proteins and Leads to the Endothelial Cell Gap

**FIGURE 4:**
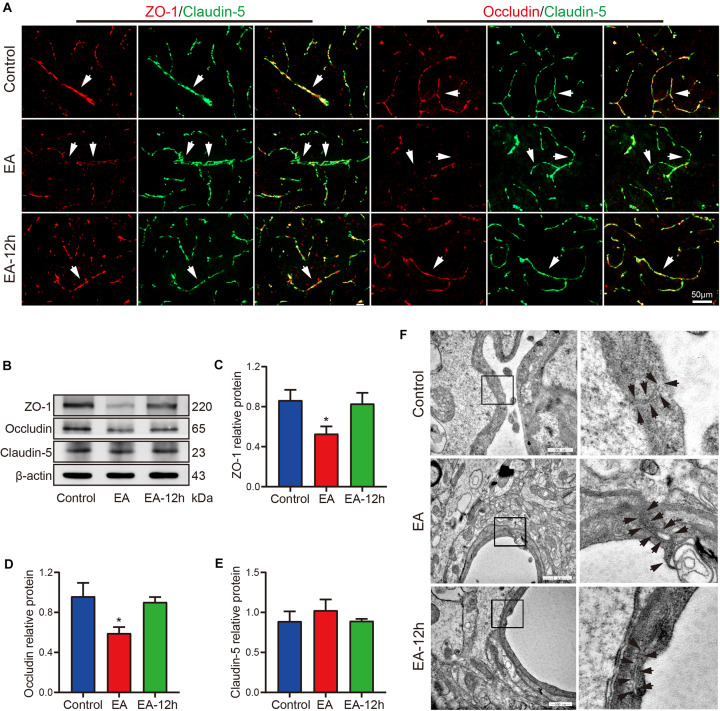
Effect of EA on tight junctions. **(A)** Changes in ZO-1, occludin, and claudin-5 expression in the control group, EA group, and EA-12 h group were observed under a fluorescence microscope, white arrows point to change places. **(B)** Western blot images illustrate the expression of ZO-1, occludin, and claudin-5 in each group. **(C, D)** The western blot data show that the expression of ZO-1 and occludin decreased after EA, *p < 0.05, EA vs control; expression of ZO-1 and occludin restore within 12 h after EA; *n* = 6/group. **(E)** The western blot data show that the expression of claudin-5 had no statistical difference in these three groups. **(F)** Changes in endothelial cell junctions in the control, EA, and EA-12 h groups were observed by TEM, black arrows point to junctions.

**FIGURE 5:**
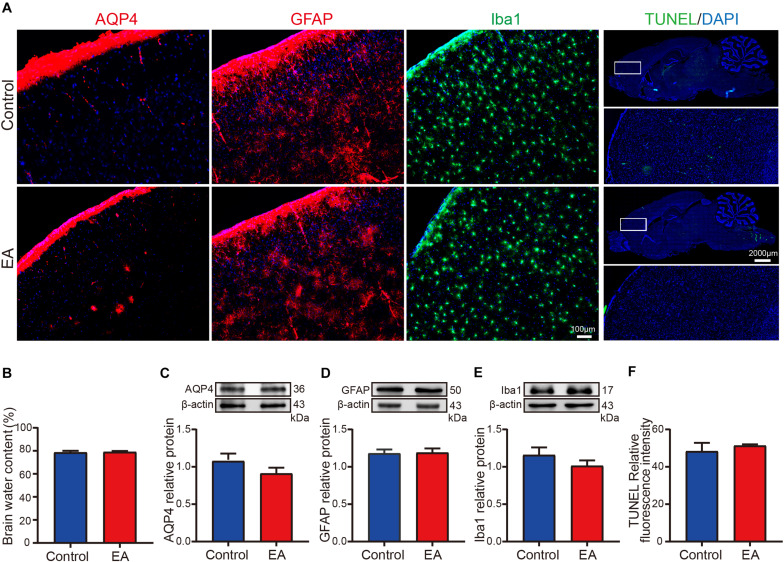
EA-induced opening of the BBB did not cause cerebral edema, inflammation, or apoptosis. **(A)** Fluorescence images showing AQP4, GFPA, Iba1, and TUNEL staining in the control group and EA group. **(B)** The statistics show that there was no significant difference in brain water content between the control group and EA group; *p* > 0.05, EA vs control; *n* = 8/group. **(C–E)** Western blot analysis shows that expression of AQP4, GFPA, and Iba1 had no significant difference; *p* > 0.05, EA vs control; *n* = 6/group. **(F)** Fluorescence analysis of apoptosis levels had no significant difference between the control and EA groups; *p* > 0.05, EA vs control; *n* = 4/group.

EA activated neurons lead to vasodilation, does it affect endothelial cell junction, will the tight junction protein expression change. We find that EA modified the expression levels of endothelial tight junction proteins. Figure 4A shows fluorescence co-staining of ZO-1 and claudin-5, occludin and claudin-5. The control group and EA-12 h group exhibited more complete and continuous expression of ZO-1 and occludin on blood vessels than the EA group, indicating that EA reduced the integrity of ZO-1 and occludin expression on blood vessels. However, there is no significant difference in the expression of claudin-5 in these three groups, indicating that EA may not affect the integrity of claudin-5 expression on blood vessels. Western blot analysis showed that EA significantly decreased ZO-1expression in the cerebral cortex (*p* < 0.05) (Figures 4B,C), and significantly decreased occludin expression in the cerebral cortex (*p* < 0.05) (Figures 4B,D), but didn’t significantly decrease claudin expression in the cerebral cortex (*p* > 0.05) (Figures 4B,E). Transmission electron microscopy showed capillary lumen, the black arrows in Figure 4F indicate endothelial cell junctions. It can be seen that there was a gap in the endothelial cells junction and an irregular endothelium, with protrusion of lipping into the lumen in the EA group. While capillaries in the control group and EA-12 h group exhibit a smooth, continuous lining endothelium, and TJ between endothelial cells are well approximated with no significant gapping. Figure 5 suggests that EA may reduce the expression of ZO-1and occludin and cause gaps between brain microvascular endothelial cells, resulting in increased permeability of the BBB, but ZO-1, occludin, and junctions can restore within 12 h after EA.

### EA-Induced Opening of the BBB Does Not Cause Obvious Negative Effects

Finally, we used the EA parameters previously used in this experiment to test whether EA can cause cerebral edema, inflammation, or apoptosis. The effects of EA on brain water content, an indicator of brain edema, were measured. Figure 5B shows the brain water content of the control and EA group, there were no significant differences (*p* > 0.05) between the EA group and the control group. AQP4, a water channel expressed on astrocytic endfeet in the brain related to brain water content, also shows no significant differences (*p* > 0.05) between the EA group and the control group (Figures 5A,C). Figure 5A shows fluorescence images of GFAP and Iba1 in the control group and EA group, and TUNEL was expressed at very low levels in the control group and the EA group. The expression of GFAP and Iba1, markers for astrocyte and microglia, respectively, as shown in Figures 5D,E, were both had no significantly increased in the EA group and the control group. Figure 5F shows that the level of apoptosis was not significantly different between the control and EA groups (*p* > 0.05) by DeadEnd^TM^ Fluorometric TUNEL system analysis. Figure 5 suggests that EA stimulation at specific parameters does not cause brain edema, inflammation of glial cell activation, or brain cell apoptosis.

## Discussion

Previous study found that the frequency, intensity, and stimulation time of EA affect its ability to open the BBB, we used a stimulation intensity of 3 mA, which was found to be safe and effective in a previous study, to evaluate the commonly used EA frequencies and stimulation time. The results showed that the frequency of 2/100 Hz on opening the BBB was better than that of EA at other frequencies. 2/100 Hz means that the 2 Hz (low-frequency) and the 100 Hz (high-frequency) exchange output. Exploratory work in human patients demonstrates a frequency-dependent variability of response in autonomic activation, and vascular diameter at various frequency stimulation frequencies (Schytz et al., 2013), and 2/100 Hz (alternating frequency), compared to a fixed frequency of 2 or 100 Hz, is not easy to make the organism adaptable and achieve a relatively continuous and stable stimulation (Cui and Ding, 2016), these may be the some of the reasons why the 2/100 Hz effect is better than other frequencies. We observed that EA can open BBB within 15 min, and keep the BBB persistently open during stimulation. So, a stimulation duration of 40 min used in this study was selected according to effectiveness, and the duration of EA usually used in the clinic. The effect on permeability is weaken immediately after EA stimulation removed and BBB completely closed finally, thus, EA-induced opening of the BBB is transient.

There are two main ways that the BBB can be opened; one is inhibiting efflux transporters, and the other is altering TJ between cells because transport across the BBB is strictly limited by both physical (TJ) and metabolic barriers (enzymes and diverse transport systems) (Blanchette and Daneman, 2015). The opening of the BBB by EA may be related to the presence of gaps at the interendothelial junctions caused by regulation of the NVU and a decrease in tight junction proteins expression. We found that the level of cerebral blood perfusion increased significantly during EA. The NVU responds to physiological stimuli, facilitating the activity-dependent regulation of vascular permeability, regulating cerebral blood flow, and activating the neuroimmune response to maintain CNS homeostasis (Sweeney et al., 2016). At the same time, c-fos in the cerebral cortex was significantly increased after EA. Fos is the protein product of the immediate-early gene c-fos, an increase in c-fos mRNA and/or protein levels is induced by neuronal activation *in vivo* (Herrera and Robertson, 1996). Thus, EA activated cortical neurons. We also found that the expression of SP increased. Substance P activates adenylyl cyclase producing cyclic adenosine monophosphate, which activates calcium ATPase, reduces intracellular calcium levels and promotes relaxation and dilation of vascular smooth muscle, and leading to cell retraction and the formation of gaps between endothelial cells, a consequence of these changes is the flux of plasma proteins from the vascular lumen into the interstitial space (Mistrova et al., 2016). But Substance P can just last from seconds to minutes in the extracellular fluid of tissue cells (Suvas, 2017). As the amount of cerebral blood flow perfusion increased significantly during EA, EA may act on endothelial cells by regulating some substances in the NVU to enlarge TJ producing gaps in endothelial cells. At the TJ, ZO-1 is associated through its first PDZ domain to the carboxyl terminal end of claudins and by its GK module to occludin, both occludin and claudins are integral proteins capable of interacting adhesively with complementary molecules on adjacent cells and of co-polymerizing laterally to maintain the basic form of TJ (González-Mariscal et al., 2003; Van Itallie and Anderson, 2014). Although the TJ strands might be composed of proteins, the structure is sensitive to rapid changes in its lipidic environment (González-Mariscal et al., 2003). We found that there was a decrease in tight junction protein expression, particularly ZO-1 and occludin, during EA stimulation, but the expression of claudin-5 has not changed significantly. Similarly, there has one study about sleep loss impairing BBB function shown that the changes of occludin and claudin-5 in BBB leakage were inconsistent (Medina-Flores et al., 2020). This could because claudins are important in establishing the tight junction pore pathway, and recent findings have shown that ZO-1 and occludin are important in the leak pathway (Shen et al., 2011).

AQP4, a critical component of an integrated water and potassium homeostasis (Yukutake and Yasui, 2010), the expression has not changed after EA. Also, we found that EA stimulation at these parameters did not cause brain edema, which usually occurs after destruction of the BBB (Michinaga and Koyama, 2017). Neuroglia, especially microglia and astrocytes, provide dynamicity to the brain. Activation of these glial cells is a major component of the neuroinflammatory responses underlying brain injury and neurodegeneration (Jha et al., 2016). GFAP and Iba1 did not increase significantly after EA, indicating that EA did not activate glial cells to cause inflammation. DNA breakage is an important biological indicator of apoptotic cells, so we used the DeadEnd^TM^ Fluorometric TUNEL system to detect apoptosis (Lee and McKinnon, 2009). The results showed that EA did not cause brain cell apoptosis, which occurs after overstimulation. These observations may have been related to the immediate closure of the BBB and an appropriate level of EA stimulation.

Therefore, we believe that 2/100 Hz EA at GV20 and GV26 for 40 min can effectively and reversibly open the BBB, possibly because this stimulation method activates neurons in the brain to cause SP release, which acts on brain vascular endothelial cells, and tight junction proteins expression decrease, forms wide gaps at the interendothelial junctions increasing permeability. When EA stimulation was stopped, SP in the brain tissue degraded rapidly, the structure of the endothelial cells slowly recovered, simultaneously, with the expression of ZO-1 and occludin gradually recovered, the vascular endothelial cell gap decreased and the BBB basically recovered. However, because of the inability to recover quickly and completely, a small amount of EB penetrated the BBB in the EA-0 h group and EA-5 h group, but the BBB completely closed within 12 h and does not cause an obvious negative effect.

Since we aimed to increase the permeability of the BBB when it was stable in the intact brain, ideal best parameters for inducing BBB opening in pathological models were not identified. Therefore, our future research direction will focus on the effectiveness of specific parameters of EA treatment for some pathological models, and the transport of molecules such as NGF or a drug to evaluate different stimulation parameters. To better visualize the process about how EA open the BBB, we will study to use dynamic real-time monitoring methods such as two-photon imaging (Zheng et al., 2019) to clarify the specific time window during which EA increases BBB permeability, and accurately control BBB permeability. Our evaluation EA-induced enhancement of BBB the permeability and BBB restoration after EA was preliminary, whether astrocytes, pericytes, enzymes, and diverse transport systems are also involved in regulating the effects of EA is waiting to continue research, the mechanism how EA affects the tight junction proteins also needs further more study.

## Conclusion

EA at 2/100 Hz and 3 mA at the GV20 and GV26 acupoints for 40 min can effectively enhance BBB permeability in the rat, and the BBB completely closes within 12 h after EA stimulation is removed. Therefore, specific parameters of EA can effectively, safely, and conveniently enhance BBB permeability. The mechanism by which BBB permeability is enhanced may be related to disrupting interendothelial TJ to forming endothelial cell gaps caused by the activation of neurons releasing SP, and a decrease in ZO-1 and occludin expression. Transiently enhancing the permeability of the BBB with EA at specific parameters may allow the delivery of more types of medications to the brain to treat brain disorders. Further investigation about the mechanistic of this method and its potential clinical applications is needed.

## Data Availability Statement

The raw data supporting the conclusions of this article will be made available by the authors, without undue reservation.

## Ethics Statement

The animal study was reviewed and approved by the Institutional Animal Care and Use Committee of Zhejiang Chinese Medical University.

## Author Contributions

SZ, JZ, and XL conceived and designed the experiments. PG, YZ, and XM performed the experiments. HW and LG analyzed the data. SZ and PG drafted the manuscript. XL and JZ revised the manuscript. All authors contributed to the article and approved the submitted version.

## Conflict of Interest

The authors declare that the research was conducted in the absence of any commercial or financial relationships that could be construed as a potential conflict of interest.
